# Recent progress of nanomedicine in the treatment of Alzheimer’s disease

**DOI:** 10.3389/fcell.2023.1228679

**Published:** 2023-06-29

**Authors:** Liqiang Hu, Yiran Tao, Yanjiao Jiang, Feng Qin

**Affiliations:** ^1^ Mental Health Center and West China-California Research Center for Predictive Intervention Medicine, West China Hospital, Sichuan University, Chengdu, China; ^2^ College of Life Science and Technology, Beijing University of Chemical Technology, Beijing, China; ^3^ Andrology Laboratory, West China Hospital, Sichuan University, Chengdu, China; ^4^ National Chengdu Center for Safety Evaluation of Drugs, State Key Laboratory of Biotherapy, West China Hospital, Sichuan University, Chengdu, China

**Keywords:** Alzheimer’s disease, nanomedicine, blood-brain barrier, nanoparticles, bioavailability

## Abstract

Alzheimer’s disease (AD) is the most common cause of memory disruption in elderly subjects, with the prevalence continuing to rise mainly because of the aging world population. Unfortunately, no efficient therapy is currently available for the AD treatment, due to low drug potency and several challenges to delivery, including low bioavailability and the impediments of the blood-brain barrier. Recently, nanomedicine has gained considerable attention among researchers all over the world and shown promising developments in AD treatment. A wide range of nano-carriers, such as polymer nanoparticles, liposomes, solid lipid nanoparticles, dendritic nanoparticles, biomimetic nanoparticles, magnetic nanoparticles, *etc.*, have been adapted to develop successful new treatment strategies. This review comprehensively summarizes the recent advances of different nanomedicine for their efficacy in pre-clinical studies. Finally, some insights and future research directions are proposed. This review can provide useful information to guide the future design and evaluation of nanomedicine in AD treatment.

## 1 Introduction

Alzheimer’s disease (AD) is an irreversible neurodegenerative disorder that causes a progressive decline in cognitive function, including memory loss, language disorders, and judgment and comprehension impairments (Masters et al., 2015; Qin et al., 2022). According to the World Alzheimer’s Disease Report 2021, there are currently around 50 million patients with AD worldwide. However, due to the growing population of aging people and the average lifespan of humans, this number is expected to increase to 78 million by 2030 (Nandi et al., 2022). AD is China’s fifth leading cause of death (Ren et al., 2022). According to the China Alzheimer Disease Report 2021, around 13.14 million patients had AD in China, and the standard prevalence was 7.88% (Ren et al., 2022). As a result, AD has become a significant healthcare challenge, with up to 0.32 million deaths in China (1.62 million deaths worldwide, accounting for 19.8% in China).

The pathological signs of AD are diverse and complex. The number of studies examining the cause and progression of AD has never stopped, since the German neurologist and psychiatrist Alois Alzheimer initially described the condition in 1907 (Breijyeh and Karaman, 2020). Although the pathogenesis of AD is still unknown, there are three accepted hypotheses for the pathological cause of AD, including the amyloid-beta (Aβ) plaque hypothesis, the Tau hyperphosphorylation hypothesis, and neuroinflammation (Busche and Hyman, 2020; Webers et al., 2020). According to the Aβ plaque hypothesis, AD is pathologically characterized by an excessive accumulation of Aβ plaques at different brain sites. Either there is an increase in Aβ plaques or an aggregation-prone form of Aβ plaques. According to the Tau hypothesis, AD is pathologically characterized by abnormally high levels of hyperphosphorylated and misfolded Tau protein that accumulate in the brain and form neurofibrillary tangles (NETs). Aβ deposition initiated a spectrum of microglia-activated neuroinflammation, and neuroinflammation is one of the key signs of AD (Thakur et al., 2023). In addition, the key signs of AD also include abnormal neurotransmitter metabolism, loss of neurons and synapses, autophagy dysfunction, and so on (Wong et al., 2020a).

Currently, no drugs on the market can stop or reverse AD progression (Breijyeh and Karaman, 2020). The acetylcholinesterase inhibitors donepezil, rivastigmine, galantamine, huperzine A, and the N-methyl-D-aspartate receptor antagonist memantine hydrochloride, which are the current clinical drugs for AD that can only temporarily relieve the cognitive symptoms of patients (Breijyeh and Karaman, 2020). Several new AD drugs have been terminated in clinical trials, including sodium oligomannate (launched in November 2019 and terminated in an international phase III clinical trial in 2022), anti-amyloid antibody aducanumab (launched in June 2021 and terminated in Europe in 2022), and gantenerumab (produced negative phase III clinical data at the end of 2022) (Bateman et al., 2022; Yeo-The and Tang, 2023). Fortunately, the monoclonal antibody (mAb), known as lecanemab, which binds to Aβ soluble protofibrils, achieved promising outcomes in its global phase III clinical trial in September 2022 (Mead and Fox, 2023). The U.S. Food and Drug Administration (FDA) approved this mAb in January 2023, through the Accelerated Approval pathway for treating AD (van Dyck et al., 2023). However, because the exact cause of AD has not yet been identified, the disease is still incurable, and most of the above therapeutic drugs can only delay its progression.

Studies found that AD drug development fails up to 99.6% between 2002 and 2012 (Elmaleh et al., 2019; Oxford et al., 2020). The blood-brain barrier (BBB) is a physical barrier that regulates how drugs enter and exits the central nervous system (CNS). BBB is one of the most crucial defense mechanisms of the CNS, and it is another significant factor hindering the development of AD drugs (Pardridge, 2019; Qin et al., 2020). Traditionally, the BBB has been difficult for almost all macromolecular drugs, including peptides, mAb, polyclonal antibodies, recombinant proteins, RNA drugs and gene drugs, and >98% of small-molecular drugs cannot do so (Pardridge, 2019). Therefore, a critical issue in AD drug development is to find the effective drug delivery strategies. Novel strategies are urgently required to overcome BBB issues, thereby increasing the possibility of success in drug development programs, considering AD drug development’s extremely high failure rate.

As nanotechnology advances quickly, several nanomaterials can assist macromolecular drugs and small-molecular drugs in penetrating through the BBB and successfully delivering them to brain, which is important for developing new drugs and treating CNS diseases (Wu et al., 2019; Jagaran and Singh, 2021; Zeng et al., 2021). More than 80 nanomedicine drugs have received worldwide regulatory approval, mainly antitumor drugs, including doxorubicin liposomes, paclitaxel albumin nanoparticles, paclitaxel polymer micelles, *etc.* (Ahmed and Qaisar, 2022). Nanomedicines can be loaded with macromolecular drugs and small-molecular drugs to successfully cross the BBB through masking, encapsulating, and embedding (Xu et al., 2019; Fan et al., 2022). Additionally, nanomedicine can be modified and grafted onto macromolecular drugs and small-molecular drugs to ensure safe targeting and controlled release. It has the benefits of a long half-life, strong targeting, a high drug loading rate, good biocompatibility, high bioavailability, and low systemic toxicity (Vissers et al., 2019; Faria et al., 2023). This review summarizes the pathogenesis of AD, the types and properties of nanomedicine, the recent advances of different nanomedicine for their efficacy in treating AD, and proposes some insights and future research directions. This review can provide useful information to guide the future design and evaluation of nanomedicine in AD treatment.

## 2 Pathogenesis of AD

**FIGURE 1:**
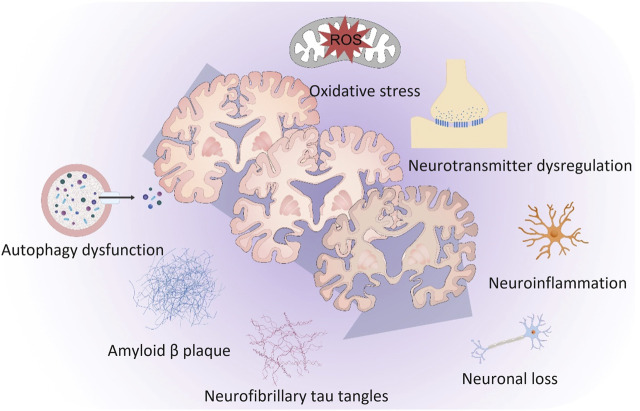
The principal factors responsible for the progression of Alzheimer’s disease. Amyloid-β plaques, neurofibrillary tau tangles and microglia-activated neuroinflammation are considered the three major pathological hallmarks of Alzheimer’s disease. Other important factors related to Alzheimer’s disease were oxidative stress, neurotransmitter dysregulation, autophagy dysfunction and neuronal loss.

The pathogenesis of AD is extremely complicated and multifactorial. The principal factors responsible for the progression of AD are shown in Figure 1. Aβ plaques, neurofibrillary tau tangles and microglia-activated neuroinflammation are considered the three major pathological hallmarks of AD. Other important factors related to AD were oxidative stress, neurotransmitter dysregulation, autophagy dysfunction and neuronal loss.

### 2.1 Aβ plaque hypothesis

The amyloid cascade hypothesis, the most recognized hypothesis to date, was proposed by British scientists Hardy and Higgins (1992). The β-amyloid precursor protein (APP) is converted into the polypeptide Aβ, which has 39–43 residues, by β-secretase and γ-secretase. Aβ exists in the brain in multiple forms, including soluble monomers, oligomers, protofibrils, and insoluble Aβ fibers in amyloid plaques (Hampel et al., 2021). Additionally, amyloid oligomers are soluble and spread throughout the brain. The two Aβ isoforms that contain 40 residues each, Aβ40 and Aβ42 are thought to be the most significant among all isoforms. The two additional residues at the C-terminus of Aβ42 are the only difference in amino acid residues between these two isoforms. However, they differ in metabolism, aggregation mechanisms, and toxicities. Particularly, Aβ40 is more likely to aggregate and form oligomers, with strong neurotoxicity, whereas Aβ40 has higher solubility and easier to remove cells. Moreover, the abundance of Aβ42 is the highest in amyloid plaques of brain samples of patients with AD (Lesné et al., 2006).

The Aβ plaque hypothesis, which suggests that Aβ protein accumulation in the brain is a key factor in AD progression, is the foundation for many drugs developed today. One of the most promising AD treatments includes immunotherapies focused on Aβ, which include strategies to decrease Aβ production, increase Aβ clearance, and inhibit Aβ aggregation. A novel fusion protein called αAβ-Gas6, developed by Jung and his team, can successfully promote Aβ clearance because it works through a different mechanism than Aβ-based mAbs (Jung et al., 2022). In addition, αAβ-Gas6 eliminated Aβ plaques in AD models without causing the neurotoxic inflammatory side effects associated with a conventional antibody treatment.

However, there are numerous negative trials of Aβ-targeting drugs for mild to moderate AD over the past 20 years, and the limited efficacy and no benefit also was observed in many trials, which have raised the question of whether Aβ is the right target (Rafii and Aisen, 2023). Recently, Aβ-targeting drugs has made substantial progress. A systematic review shows that aducanumab reduced brain Aβ plaques in a time- and dose-dependent manner, and aducanumab reduced the severity of clinical dementia in the high-dose treated group (Rahman et al., 2023). In addition, lecanemab was found to produce marked lowering of Aβ plaques based on evidence derived from amyloid positron emission tomography, and lecanemab shows “clinical benefit” for the AD treatment, paving the way for the medication to be considered for full FDA approval (Mahase, 2023; van Dyck et al., 2023).

### 2.2 Tau hyperphosphorylation hypothesis

Along with Aβ and cerebrovascular amyloid angiopathy, neurofibrillary tangles (NFTs) and neuropil threads are neuropathological confirmations in diagnosing AD. The aberrant intraneuronal Tau protein, which is highly neurotoxic and impairs cognition, makes up the entire structure of NFTs. An overview of the discovery of Tau proteins dates back to 1974 when American scientist Iqbal et al. isolated NFT and paired helical filament (PHF) proteins from the brains of patients with AD for the first time (Iqbal et al., 1974). In the following year, Weingarten et al. (1975) isolated Tau protein from pig brains. Then, Grundke-Iqbal et al. (1986) found that PHFs consist of Tau protein, which is significantly more phosphorylated than normal human brain Tau. Tau protein is a microtubule-associated protein that is highly soluble and abundant in the neurons of the CNS. It primarily affects the distal end of axons, preserving the stability and flexibility of microtubules while also regulating axonal transport and promoting actin filament formation (Venkatramani and Panda, 2019). Normal Tau carries multiple phosphate groups in its microtubule assembly domain, but phosphorylation of tau reduces its affinity for microtubules and begins to aggregate in its hyperphosphorylated form (Mohammadi et al., 2023). Elevated phosphorylation and aggregation of Tau are widely considered pathological hallmarks in AD (Wegmann et al., 2021).

Dr. Mielke found that peripheral blood phosphorylated Tau at threonine-181 (P-Tau 181) and phosphorylated Tau at threonine-217 (P-Tau 217) levels are reliable indicators of cerebral amyloid lesions and can be used as blood-based biomarkers to distinguish AD from other neurodegenerative diseases (Mielke et al., 2022). Stabilizing the structure of tubulin, inhibiting the phosphorylation and aggregation of Tau, and promoting the degradation of aggregated Tau are the major steps in developing drugs that target Tau. Fang et al. found that sildenafil has neuroprotective and neurorestorative roles by promoting neurite growth and reducing aberrant Tau protein phosphorylation (Fang et al., 2021).

### 2.3 Neuroinflammation

Neuroinflammation is another distinguishing hallmark of the brain in AD patients, along with Aβ plaques and NFTs, and it is also thought to play a role in the progression of AD (Si et al., 2023). Study has shown that the serum levels of inflammatory cytokines, such as interleukin-6 (IL-6), IL-1β, tumor necrosis factor α (TNF-α), and interferon-γ, are significantly higher in patients with AD than in healthy individuals (Demirci et al., 2017). The risk of AD is increased by chronic inflammation in the brain, which is mediated by interactions between the immune system and the CNS. Before Aβ plaque deposition, neuroinflammation occurs in the very early stages of AD. Recent studies suggest that as the disease advances, neuroinflammation promotes the synthesis of new Aβ, and increased Aβ levels in the brain can induce and exacerbate neuroinflammation (Bronzuoli et al., 2016; Yang, 2019). This leads to a vicious cycle between Aβ accumulation and neuroinflammation.

The resident brain macrophages known as microglia, detected in certain brain areas in patients with AD, have been found to promote phagocytosis to monitor and protect neural tissue. Hexokinase 2 (HK2), a glycolytic enzyme, is shown to be highly upregulated in microglia in a mouse model of AD (Leng et al., 2022). The increased activity of HK2 resulted in insufficient energy production in these cells, decreasing microglial phagocytosis. However, HK2 inhibition causes a switch from glucose to lipid metabolism to ensure efficient energy, leading to microglia with strong phagocytic activity to clear Aβ plaques and improve cognitive function (Choi and Mook-Jung, 2022; Leng et al., 2022). However, there is a debate concerning the role of microglia in AD, and when these cells lose their ability to maintain homeostasis, they may have harmful effects. The pyrin-domain containing 3 (NLRP3) inflammasome is one of the multiprotein complexes that microglia can produce. When NLRP3 is activated, it can cleave the downstream inflammatory factor IL-1β from the precursor to the mature state and release it into the extracellular space, which causes neuroinflammation (Heneka et al., 2018). Further studies have found that Aβ-induced Tau lesions depend on the NLRP3 inflammasome, and the NLRP3 inflammasome plays a role in the development of Tau lesions and AD. IL-1β is also highly expressed in the cortex of patients with frontotemporal dementia, suggesting inflammasome activation.

Following the interferon-induced transmembrane protein 3 (IFITM3) in aging mice compared to healthy mice, another study found that IFITM3 is upregulated in tissue samples from patients with advanced AD (Hur et al., 2020). Anti-inflammatory drug administration that can lower IFITM3 levels also reduces the production of Aβ, delaying the onset and progression of AD. IL-3 is a crucial mediator of astrocyte-microglia crosstalk and a node of AD therapeutic intervention, and the study of Harvard University discovered that it had a protective effect in an AD mouse model (McAlpine et al., 2021). These findings suggest that regulating neuroinflammation might be a novel treatment option for AD.

### 2.4 Dysregulation of neurotransmitters

In the brains of patients with AD, there is a general decrease in the levels of neurotransmitters, particularly those involved in learning and memory, such as the acetylcholine (Ach) system, amino acids, monoamine system, neuropeptides, and other neurotransmitters (such as γ-aminobutyric acid, glutamate, epinephrine, *etc.*). Previous study has confirmed that Ach and choline acetyltransferase levels in the hippocampus and cortex of patients with AD are significantly decreased (DeKosky et al., 2002). Current standard treatments for patients with mild-to-moderate AD include cholinesterase inhibitors such as donepezil, rivarastine, galantamine, and huperzine A. The basic mechanism is to increase the concentration of Ach in the brain and prevent cholinesterase (Snowden et al., 2019). Studies have shown that elderly patients with cholinesterase inhibitors can also improve cognitive function and promote the release of dopamine, norepinephrine, epinephrine, and other neurotransmitters in the brain (Wynn and Cummings, 2004). However, cholinesterase inhibitors can cause side effects such as nausea, vomiting, and diarrhea (Hogan, 2014).

### 2.5 Oxidative stress

There is growing evidence that oxidative stress plays a key role in the pathogenesis of AD (Sultana and Butterfield, 2010). Reactive oxygen species (ROS) can cause the oxidation of intracellular biological macromolecules (such as nucleic acids, membrane lipids, and cellular proteins), which can negatively impact synaptic activity, damage nerve cells, and result in cognitive dysfunction. Loss of mitochondrial function, altered metal homeostasis, and decreased antioxidant capacity can increase ROS production. ROS also promotes aberrant Aβ deposition and Tau hyperphosphorylation, which aggravates mitochondrial dysfunction and ROS production, resulting in AD and creating a vicious cycle (Kent et al., 2020; Wang et al., 2020). Therefore, inhibiting mitochondrial ROS overproduction, metal ion chelation therapy, and increasing antioxidant capacity can improve the symptoms of AD.

### 2.6 Others

Mitophagy is an autophagy mechanism that selectively eliminates defective mitochondria, preserving cellular homeostasis and energy metabolism in cells. Early cellular homeostasis in AD involves mitochondrial damage, and cell ROS levels result in abnormal mitophagy and autophagy. A recent study has shown a previously unreported pattern of autophagic stress, known as poisonous anthos flower, associated with impaired neuron autophagy and present in AD brains (Lee et al., 2022). Although the molecular basis for mitophagy and autophagy dysfunction in AD is unknown, this pattern of autophagic stress has been linked to AD (Lee and Nixon, 2022). Additionally, using imaging and histochemical methods, they showed that the neurons are the source of the great majority of senile plaques in AD mice models, leading to a re-evaluation of the generally understood sequence of events in plaque formation in AD (Lee et al., 2022).

Recent studies show that the most common genetic risk factor for AD is the lipid and cholesterol gene apolipoprotein E4 (APOE4) (Simon et al., 2012; Ryu et al., 2019; Safieh et al., 2019). One copy of the APOE4 allele increases the risk of getting AD by three to four times, while two copies can increase the risk by 8–12 times (Simon et al., 2012). According to the studies, the APOE4 allele is present in 50%–60% of patients with AD (Abushakra et al., 2016; Ryu et al., 2019). APOE4 drastically changed the signaling pathways involved in cholesterol homeostasis and transport, hampered myelination, and impacted cognitive function in mice. In mice with APOE4, pharmacological intervention to lessen this effect enhanced learning and memory. This study provides a prospective treatment strategy for AD by demonstrating a functional relationship between APOE4, cholesterol, myelination, and memory (Blanchard et al., 2022). In addition, the APOE4 gene causes glia-specific cellular and cell-free dysregulation, which raises the risk for AD (Tcw et al., 2022). The studies mentioned above present a new strategy of treating AD.

## 3 Types and properties of nanomedicine

Developing anti-AD drugs is challenging due to the complex etiology and unclear pathogenesis, and getting adequate drug delivery across the BBB is another major challenge (Roy et al., 2022). However, nanomedicine-based drug delivery strategies provide complementary opportunities for AD. Increasing efforts have been made in recent years to deliver drugs to the brain, some of which have entered clinical trials. Nanomedicine is one of the effective methods for delivering drugs to cross the BBB. Additionally, nanomedicines can improve the biodistribution and pharmacokinetics of drugs through oral, intravenous, and intranasal delivery. Therefore, nanomedicines have been thoroughly researched for the treatment of AD (Saraiva et al., 2016). However, inorganic materials are difficult to break down, which may result in toxicity and restrict clinical applications (Li et al., 2021), include metal nanoparticles (NPs) and carbon nanotubes (CNTs). In contrast, liposomal and polymeric NPs are widely used in approved nanomedicines due to their high drug loading, good biocompatibility, and nontoxicity (Hettiarachchi et al., 2019). The ideal AD nanomedicine typically possesses the following characteristics: particle size 10–100 nm, BBB penetration, high drug loading, long action time, good drug stability, nontoxicity, biodegradability, simple fabrication process, noninvasive administration, low cost, and stable storage (Jagaran and Singh, 2021).

**FIGURE 2:**
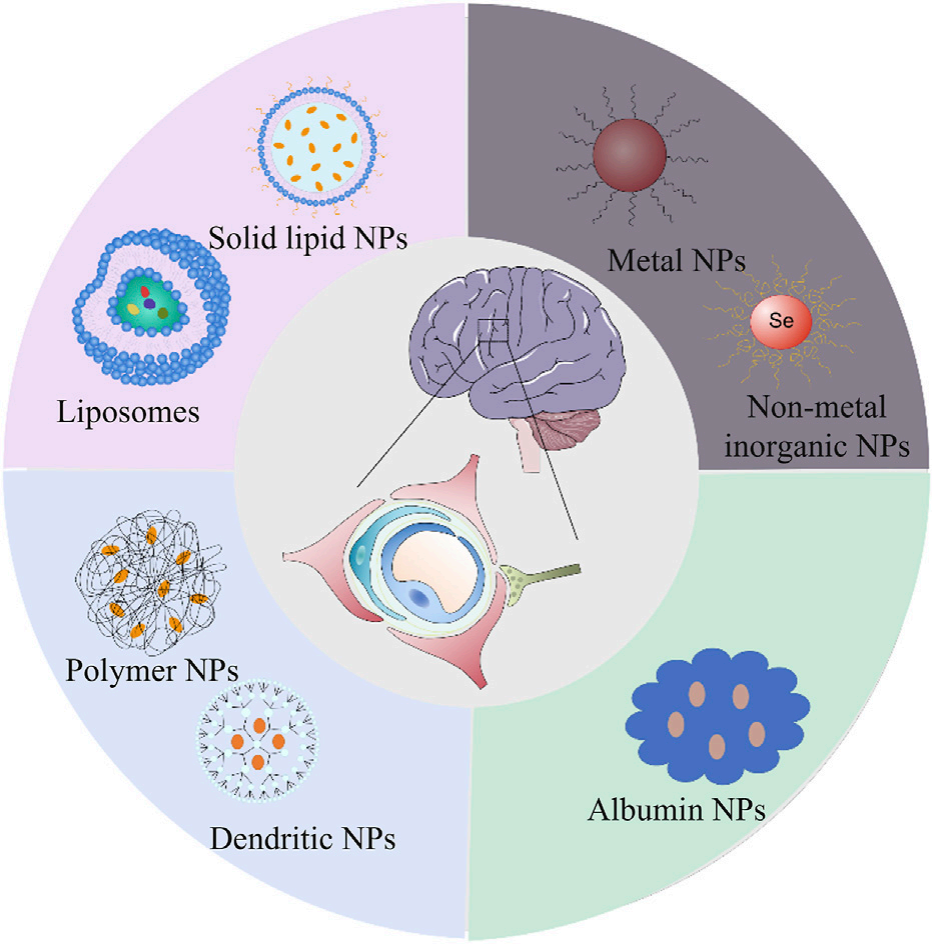
Schematic representation of the structures of the most commonly used nanomedicine types for Alzheimer’s disease: lipid (liposomes and solid lipid), organic (dendritic and polymer), inorganic (metal and non-metal inorganic) and biomimetic (albumin).

In 1971, Gregoriadis discovered that using liposomes as drug carriers could reduce the toxic effects of drugs and increase their ability to work as intended (Gregoriadis and Ryman, 1971). Liposomal amphotericin B, the first nanomedicine, was introduced in the Irish market in 1990. After more than 50 years of rapid development, nanomedicine is currently a topic of interest in treating AD. Liposome drugs were listed earlier and made up most of the currently commercialized nanocarrier drugs, such as amphotericin B liposomes, daunorubicin liposomes, doxorubicin liposomes, paclitaxel liposomes, *etc.* (Crommelin et al., 2020). Followed by microspheres, common ones include octreotide, risperidone, leuprolide, *etc.* Many nanoparticles for AD have been studied and developed with the continuous innovation of drug delivery systems. The reported nanomedicines of AD mainly include liposomes, solid lipids NPs, polymer NPs, dendritic NPs, albumin NPs, metal NPs, non-metal inorganic NPs, *etc.* (shown in Figure 2). However, only a few of these NPs have been approved for clinical use, and most are still in the fundamental research stage (Mitchell et al., 2021). Table 1 highlights *in vivo* studies of the nanomedicines for AD therapy from 2019 to 2022.

**TABLE 1:** *In vivo* studies of nanomedicines for Alzheimer’s Disease.

	Main materials	Target hypothesis	Loaded-drugs	*In-vivo* model	Results	References
**Polymer NPs**						
	PLGA	Aβ	None	5xFAD AD mice	Reduce Aβ plaques in PLGA-treated 5xFAD mice	Anand et al. (2022)
	PLGA	Aβ	None	C57 mice	Mediate the clearance of Aβ1–42 in the brain	Paul et al. (2022)
	PLGA	Neuroinflammation	Anti-TRAIL monoclonal antibody	3xTg AD mice	Increase the presence of anti-TRAIL antibody in the brain	Musumeci et al. (2022)
	PLLA and PLGA	AChE	Galantamine	Wistar rat	Increase the uptake of galantamine by neurons in the entire hippocampal formation	Nanaki et al. (2020)
	PLA-PEG	Aβ	Bies virus protein 2, rifampicin, gadolinium	APP/PS1 transgenic mice	Reduce Aβ plaques in hippocampus and cortex, repair the synapse structure	Zhou et al. (2022)
	Chitosan	Aβ, oxidative stress	Tanshinone IIA	*Caenorhabditis elegans* model of AD	Increase the concentration of Tanshinone IIA, extend the lifespan and promote reproduction of *Caenorhabditis elegans*	Zhang et al. (2022)
	Chitosan	Aβ, oxidative stress	Luteolin	STZ-induced AD mice model	Improve the acquisition of short-term memory, increase in the number of surviving neurons in all hippocampus regions	Abbas et al. (2022)
	Chitosan	Aβ	Cannabidiol	Aβ1-42 peptide-induced AD rat model	Reduce Aβ plaques, increase brain cannabinoid receptor type 1 and 2 levels	Amini & Abdolmaleki (2022)
**Liposomes**						
	DSPE-PEG-MAN, DSPE-PEG-CPP, DOPE, DOTAP, and cholesterol	Neurotrophic factors	Plasmid encoding BDNF	APP/PS1 transgenic mice	Enhance BDNF expression, reduce plaque, increase synaptic protein level	Arora et al. (2022)
**Solid lipid NPs**						
	Lecithin, stearic acid, cholesterol, Tween 80	Oxidative stress	Pomegranate extract	AlCl_3_-induced AD rat model	Increased the efficacy of active components	Almuhayawi et al. (2020)
	compritol, Tween 80, chitosan	Oxidative stress	Ferulic acid	STZ-induced AD rat model	Enhance ferulic acid concentration in brain	Saini et al. (2021b)
	Compritol^®^ 888 ATO, Lutrol F68^®^	Aβ, oxidative stress	Curcumin	TgCRND8 mice	Restore the mitochondrial functions	Campisi et al. (2022)
**Dendritic NPs**						
	Prussian blue, Polyamidoamine dendrimer, angiopep-2	Aβ, oxidative stress	None	APP/PS1 transgenic mice	Reduce neurotoxic Aβ aggregation; rescue the cognitive functions	Zhong et al. (2022)
**Albumin NPs**						
	Human albumin	Aβ, oxidative stress	Andrographolide	CRND8 transgenic mice	Decrease the oxidative stress levels; amelioration of cognitive functions	Bilia et al. (2019)
	Bovine serum albumin	Aβ	Aβ-binding peptides	Aβ-induced AD mice model	Reduce Aβ deposition and TNF-α level in brain	Zhao et al. (2019)
**High-density lipoprotein NPs**						
	Native HDL of human plasma fraction IV	AChE, Aβ	Donepezil	Aβ1-42 peptide-induced AD mice model	Inhibit AChE activity and reduce Aβ deposition in donepezil-loaded HDL treated AD mice	Zhang et al. (2019)
**Exosomes**						
	Exosomes from whole blood	Tau	Quercetin	Okadaic acid- induced AD mice model	Inhibit phosphorylated Tau-mediated neurofibrillary tangles	Qi et al. (2020)
	Exosomes derived from curcumin-treated RAW 264.7 cells	Tau	Curcumin	Okadaic acid- induced AD mice model	Inhibit Tau phosphorylation, attenuate cognitive decline	Wang et al. (2019)
	Exosomes from bone marrow of mice	Aβ, neuroinflammation	Rabies viral glycoprotein peptide	APP/PS1 transgenic mice	Reduce plaque deposition, inhibit astrocytes activation	Cui et al. (2019)
**Magnetic NPs**						
	SPIONs	Aβ, neuroinflammation, oxidative stress	Aβ oligomer-specific scFv antibody W20 and class A scavenger receptor activator XD4	APP/PS1 transgenic mice	Reduce neuroinflammation and ROS level, increase glutathione level, reduced reactive oxygen species level, rescue cognitive deficits and alleviate neuropathology	Liu et al. (2020a)
	PEGylated SPIONs	Aβ	None	Aβ1-42 peptide-induced AD rat model	Improve BDNF and p-CREB levels, inhibit Aβ aggregation, and improve learning and memory ability	Sanati et al. (2021)
	Quercetin-conjugated SPIONs	Oxidative stress	Quercetin	AlCl_3_-induced AD rat model	Anti-oxidation, anti-apoptosis, reduce the expression level of APP gene, improve cognitive function	Amanzadeh Jajin et al. (2021)
**Metallic nanoparticles**						
	Au	Aβ, oxidative stress	L- or D-glutathione	APP/PS1 transgenic mice	Decrease Aβ deposition, rescues the spatial learning and memory impairments	Hou et al. (2020)
	Ru	Tau, oxidative stress	NGF	Okadaic acid- induced AD mice model	Control NGF release, inhibit tau hyperphosphorylation, inhibit ROS production	Zhou et al. (2020)
**Non-metal Inorganic Nanoparticles**						
	Se-doped carbon quantum dots	Aβ, oxidative stress	None	AD rat model	Reduce Aβ accumulation, ROS-scavenging activity	Zhou et al. (2021)
	Se nanoparticles	Neuroinflammation, Aβ	Resveratrol	AlCl_3_-induced AD rat model	Downregulate STAT3 and IL-1β, antioxidant activity, anti-inflammatory effect	Abozaid et al. (2022)
	Sulfur nanoparticles	Neuroinflammation, oxidative stress	Quercetin	APP/PS1 transgenic mice	Reduce neuronal apoptosis, inflammatory response, calcium homeostasis imbalance, and oxidative stress	Liu et al. (2020b)
**Others**						
	Regadenoson, nitric oxide donor and YC-1	Aβ	Donepezil	AD rat model	Beneficial for synaptic plasticity and memory formation	Liu et al. (2021)
	Nanocurcumin	Oxidative stress; AChE	Curcumin	STZ-induced AD rat model	Ameliorate the behavioral, immunohistochemical and most of the neurochemical changes	Noor et al. (2022)
	Nano-Honokiol	Neuroinflammation,Aβ	Honokiol	TgCRND8 mice	Improve cognitive deficits, inhibit neuroinflammation via suppressing the levels of TNF-α, IL-6 and IL-1β in the brain, reduce Aβ deposition in the cortex and hippocampus of mice	Qu et al. (2022)
	RBC membranes, PLGA, T807	Tau	Curcumin	Okadaic acid- induced AD mice model	Reduce the total p-tau level, increase curcumin accumulation in the brain	Gao et al. (2020)

Abbreviations: Aβ, amyloid β-protein; AChE, acetylcholinesterase; AD, alzheimer’s Disease; APP/PS1, amyloid precursor protein/presenilin 1; BDNF, brain-derived neurotrophic factor; CREB, element-binding protein; HDL, high-density lipoprotein; IL-1β, interleukin-1β; NGF, nerve growth factor; PEG, polyethylene glycol; PLA-PEG, poly (l-lactide)-poly (ethylene glycol); PLGA, poly (lactide-co-glycolide); PLLA, poly (l-lactic acid); ROS, reactive oxygen species; SPIONs, superparamagnetic iron oxide nanoparticles; STAT3, signal transducer and activator of transcription; STZ, streptozocin; TRAIL, tumor necrosis factor-related apoptosis inducing ligand; 3xTg AD, the triple-transgenic mouse model of AD.

### 3.1 Polymer NPs

In a promising nanoscale drug delivery system, polymer NPs can encapsulate drugs within a biodegradable polymeric matrix or alter their surface to provide a controlled release and improved bioavailability. Polymer NPs can range from 10 to 1,000 nm. Poly (lactic-co-glycolic acid) (PLGA), polyethylene glycol (PEG), poly-L-lysine, polylactic acid (PLA), polyethyleneimine, poly (acrylate-co-alkyl acrylate) (PACA), poly (butyl cyanoacrylate), polycaprolactone, chitosan, and gelatin are commonly used to make polymer NPs (Zhang W. et al., 2021). The basic idea behind this class of nanoscale drug delivery systems is to encapsulate drugs within the matrix of the carrier material or to modify them by covalent linkage/adsorption on the surface of the carrier material. The excellent biocompatibility, high bioavailability, and controllable biodegradability of polymer NPs span a broad spectrum. The technology is currently one of the most active research areas, and has enormous potential for application in nanomedicine.

Extensive studies shows that curcumin has anti-Aβ formation, aggregation, anti-inflammatory, and antioxidant effects that can help middle-aged and older people with mild cognitive impairment improve their memory (Fan et al., 2018; Yang et al., 2018). Therefore, Fan et al. designed a brain-targeted curcumin NPs system containing PLGA-PEG combined with B6 peptide and curcumin. The results showed that compared with curcumin administration, curcumin NPs could reduce hippocampal Aβ deposition and excessive Tau protein phosphorylation, significantly improving the spatial learning and memory ability of APP/Presenilin 1 (PS1) mice (Fan et al., 2018). Additionally, Yang et al. developed bovine serum albumin and chitosan loaded with curcumin. They found that the NPs could effectively increase the penetration of drugs through the BBB, promote microglia activation, and accelerate the phagocytosis of Aβ42 (Yang et al., 2018).

Donepezil is primarily used to treat mild and moderate AD. However, the drug’s concentration in the brain is restricted due to poor penetration. Therefore, to prepare organic NPs loaded with donepenzil, Liu et al. introduced regadenoson (Reg) at the end of PEG2000. Reg-NPs could enhance drug delivery into the brain, and activate the PKG/PI3K and NO/cGMP signaling pathways. The NPs improved learning and memory in Aβ-induced AD mice as compared with donepezil at the same dose (Liu et al., 2021).

Depending on how they charge, polymer NPs can be divided into cationic, anionic, and zwitterionic polymers. Positively charged polymers are amine-containing polymers that can electrostatically adsorb or encapsulate negatively charged nucleic acids. Wang et al. constructed PEG-polydimethylaminoethyl methacrylate (cationic polymer) NPs loaded with β-site amyloid precursor protein cleaving enzyme 1 (BACE1) to treat APP/PS1 transgenic mice. According to the findings, these NPs significantly decreased BACE1 mRNA and Aβ plaques, inhibited Tau phosphorylation, and promoted hippocampal neurogenesis (Wang et al., 2018).

### 3.2 Liposomes

Liposomes are the earliest nanomedicine delivery system to proceed from concept to preclinical research and clinical application. Liposomes mainly comprise natural lipids, usually phospholipids, ionizable, cholesterol, and pegylated. Liposomes have no pharmacological activity, low toxicity, and good biodegradability, mobility, and biocompatibility (Patel et al., 2021). Therefore, they promise vehicles to deliver therapeutics, such as antitumor agents and anti-inflammatory, antibiotic, antifungal, anesthetic, and other drugs. Moreover, liposomes’ hydrophilic and hydrophobic components can encapsulate water-soluble and lipid-soluble drugs, improving their bioavailability. Several liposomal drugs have been approved for marketing worldwide. Chen et al. modified liposomes with transferrin to improve the brain delivery of α-mangostin, a polyphenolic xanthone effective in treating AD. In addition, the transferred liposomes could successfully transport α-mangostin across the BBB *in vitro* and *in vivo* (Chen et al., 2016).

Tannic acid is a multi-branched polyphenol molecule that effectively inhibits Tau aggregation. Hu et al. coated the liposomes with Tween 80 and added tannic acid to the centre of each one, tannic acid liposomes can cross the BBB and deliver drugs effectively (Hu et al., 2020). In addition to internal drug loading, phospholipids can react with the drug to synthesize the phospholipid prodrug, which can then assemble with other lipids to form liposomes with surface modification of the drug and achieve an anti-AD effect. Introducing phosphatidic acid with Aβ affinity into the liposome component can also endow the liposome with certain AD efficacy. Mancini et al. used phosphatidic acid to create liposomes (Mancini et al., 2016). The modified ApoE-derived peptide was also added to the surface of the liposomes to increase BBB permeability. The outcomes demonstrated that the generated liposomes might delay the cognitive function decline in mice. Furthermore, the liposomes were optimized for drug delivery and treatment, after administration, the area and number of Aβ plaques in the cortex and hippocampus were reduced by 29% and 31%, respectively. The above studies have shown that liposomes help drugs enter the brain, making them the most promising drug-carrier system for treating AD.

### 3.3 Solid lipid NPs (SLNs)

SLNs are solid core lipid nanocarriers, which are developed to hold both hydrophilic and lipophilic drugs (Paliwal et al., 2020). The particle size ranges between 10 and 1,000 nm, and fatty acids, fatty alcohols, and phospholipids comprise most of the components in SLNs. The drug is encapsulated or trapped in the lipid core to create a solid colloidal drug delivery system. Due to their improved physical stability, higher loading capacity, better bioavailability, and ease of large-scale production, SLNs are excellent for poorly soluble drugs (e.g., adriamycin and cyclosporine). Therefore, it has been widely used to create various dosage forms, including oral, injection, transdermal, and pulmonary delivery, and has broad prospects in developing new drugs for AD.

Ferulic acid is an active component of herbal medicines, which can inhibit Aβ aggregation and improve the viability of AD cell models *in vitro* and is considered as a potential drug for AD treatment (Mancuso and Santangelo, 2014; Kikugawa et al., 2016). However, Ferulic acid has the characteristics of poor water solubility and limited brain permeability. Therefore, using Compritol as a lipid and Tween 80 as a surfactant, Saini et al. constructed SLNs for ferulic acid. SLNs significantly improved learning and memory functions in AD rats and had an excellent nasal mucosal adhesion and penetration ability (Saini et al., 2021). Additionally, Vedagiri and Sumathi created SLNs loaded with chrysin, and found that SLNs could increase the bioavailability of chrysin and decrease the neuronal damage caused by Aβ25-35 (Vedagiri and Sumathi, 2016). Yusuf et al. created piperine-loaded SLNs in a different study. They found that SLNs could improve Aβ plaque deposition by reducing oxidative stress and cholinergic degradation in the cortex of AD rats induced by atraconic acid (Yusuf et al., 2013). Additionally, Dhawan et al. created quercetin-loaded SLNs, they found that compared to pure quercetin, SLNs significantly decreased their antioxidant capacity and prevented the neurodegeneration induced by alumina in rats (Dhawan et al., 2011). According to the studies above, SLNs can cross the BBB and increase the bioavailability of insoluble drugs, and it have good potential as nanocarriers for the treatment of AD.

### 3.4 Dendritic NPs

Highly branching globular polymers, known as dendritic NPs, often have compact three-dimensional structures with a central core and corona. They mostly use dendrimers made of polyamide-amine (PAMAM) (Janaszewska et al., 2018). Their unique three-dimensional structure gives them unique properties, such as surface modification of functional groups, spherical nanostructures, internal hydrophilic or hydrophobic cavities, and a low polydispersity index (Li et al., 2021). The internal core of dendrimers, which contains functional groups, provides space and binding capabilities for several drug molecules, including chemical compounds, peptides, genes, and other substances.

A novel class of nanocomposites, known as dendritic NPs, has been applied in AD drug development. The efficient passage of dendritic NPs through the BBB can be attributed to its lipophilicity (Kaurav et al., 2023). Modifying the surface of dendrimers will improve biocompatibility and enhance drug transport through the BBB (Zhu et al., 2019). Kumari et al. demonstrated how the preparation and synthesis of hyperbranched dendritic polymers, such as PACA, PLA, and PLGA, can inhibit fiber formation and have a specific therapeutic effect on AD (Kumari et al., 2010). PAMAM dendrimers can disrupt existing fibrils and interfere with the development of Aβ1-28 in AD brain, which can delay the progression of AD (Klajnert et al., 2006). Additionally, Klementieva et al. found that maltose (MAL)-modified polypropylamine (PPI) dendrimers can prevent Aβ1-40 fibrosis. They include PPI-G5-MAL, which can block the formation of amyloid fibers, and PPI-G4-MAL, which can reduce amyloid toxicity by aggregating fibers (Klementieva et al., 2011). Hence, dendritic NPs may have promising application prospects in treating AD.

### 3.5 Biomimetic NPs

#### 3.5.1 Albumin NPs

Albumin is the most abundant protein in plasma, with 50% of the total protein content of plasma proteins. Common albumins include ovalbumin, bovine serum albumin, human serum albumin (HAS), and recombinant HAS. Albumin is an endogenous substance and is water soluble and biocompatible. Therefore, it can be injected intravenously and metabolized *in vivo* without releasing toxic chemicals (Mariam et al., 2016). Covalently binding drugs to albumin, physically adsorbing drugs on the surface of the albumin, or encapsulating drugs inside the matrix of albumin NPs are a few examples of albumin-mediated drug delivery systems. HAS cannot cross the BBB directly, but it can be injected into the nasal cavity, by passing the BBB and entering the brain (Piazzini et al., 2019). The NPs can greatly improve drug transport across the BBB and accumulate therapeutic drugs in the brain.

A nanocomposite known as NC-KLVFF, which contains bovine serum albumin as the core and the KLVFF peptide modified on the surface, can significantly inhibit the oligomerization of Aβ and fibrosis. *In vivo,* NC-KLVEF can bind to Aβ to form a complex, which then promotes the uptake and clearance of Aβ by microglia. Simultaneously, to avoid damage to scavenger receptors caused by Aβ oligomers triggering microglia to secrete inflammatory factors, the scavenger receptor type A and CD36 on the surface of microglial cells were maintained. Furthermore, the ability of microglia to phagocytose is attenuated. It also downregulates the expression of scavenger receptors on the surface of microglia (Zhao et al., 2019).

Yang et al. created NPs using HAS as the core to encapsulate donepezil and clioquinol (a metal-ion chelating agent), and the outer-modified transmembrane brain and monosialotetrahexosyl ganglioside to increase BBB permeability and Aβ permeability (Yang et al., 2020). The results showed that surface modification of the transmembrane brain and monosialotetrahexosyl ganglioside could improve NPs’ brain entry efficiency and retention ability. In addition, clioquinol inhibited the aggregation of Aβ induced by copper and zinc and promoted the degradation of aggregated Aβ. Donepezil can alleviate the progression of the disease in many ways, reduce the inflammatory response associated with Ach in mouse microglia cells, regulate the balance of Ach, and achieve synergistic treatment.

#### 3.5.2 High-density lipoprotein (HDL) NPs

Natural HDL mainly consists of phospholipids, cholesterol, and apolipoproteins. It is easy to express *in vitro*, has good biosafety and compatibility, and possesses the structural characteristics of a hydrophilic outer layer and a hydrophobic inner layer (Damiano et al., 2013). The hydrophobic core of the prepared NPs can contain poorly soluble drugs, and the surface can also provide gene drugs with covalent site misdetermination of amphiphilicity. Furthermore, the surface of the NPs can be modified with functional phospholipids such as phosphatidic acid. Additionally, native HDL has excellent brain-targeting properties and is a promising drug delivery carrier for AD due to its high expression of lipoprotein receptors on the BBB.

It has been reported that the NPs of HDL have an affinity for Aβ, which can facilitate Aβ binding in the brain and Aβ absorption and degradation by microglia and astrocytes. In addition, the NPs can bind free Aβ in the blood, promote hepatocytes to phagocytose, degrade Aβ, and maintain the metabolic balance of Aβ in the body (Song et al., 2014). The HDL carrier possesses Aβ affinity characteristics and can target the BBB. Inspired by “HDL bionics”, Zhang et al. delivered donepezil via an apolipoprotein A-I-reconstituted HDL to enable simultaneous clearance of Aβ and inhibition of acetylcholinesterase (Zhang et al., 2019). ApoA-I can target the scavenger receptor SR-BI on the surface of the BBB to efficiently transtranscytophoresize NPs across the BBB. Since ApoA-I has a high affinity for Aβ, it can be taken up by cells, combined with Aβ to form nanocomposites, inhibit Aβ aggregation, promote metabolic clearance of Aβ through the lysosomal pathway, relieve the burden of Aβ in the brain, and inhibit the activity of acetylcholinesterase. The double-effect treatment for AD can be achieved by increasing the content of Ach at the receptor site.

Additionally, lipoprotein NPs have therapeutic effects on the brain’s microenvironment and the lesion site’s damaged nerves and blood vessels. The neurovascular unit (NVU), composed of neurons, glial cells, and blood vessels, is crucial to the brain microenvironment’s stability. Therefore, NVU impairment is a key pathogenic event in AD that is closely related to the progression of the disease and cognitive decline (Iadecola, 2017). A multifunctional lipoprotein-like nanostructure called RAP-RL, which contains an antagonist peptide (RAP) of the receptor for advanced glycation end-products (RAGE), has been developed to intervene in the progression of AD and modify the NVU (Zhang Q. et al., 2021). RAP-RL can significantly reduce the amount of Aβ accumulated in the hippocampus of APP/PS1 mice by 55.24% and the amount of Aβ accumulated in the cortex by 44.14% through the specific binding of RAP to RAGE. In addition, it can also decrease the levels of the inflammatory factors TNF- and IL-6, restore NVU integrity and function, improve cerebral blood flow, and increase learning and cognitive ability in mice.

#### 3.5.3 Exosomes

Exosomes are extracellular nanosized biovesicles that function as intercellular messengers because they contain proteins, lipids, and nucleic acids in bodily fluids. Undoubtedly, exosomes are potential bionanoparticles for drug delivery. Furthermore, exosomes are an attractive and safe platform for brain-targeted delivery because they are small, easily cross the BBB, and have low immunogenicity as cell-derived vesicles. However, whether or how they cross the BBB remains unclear (Lin et al., 2020).

Several exosome-related drugs have shown therapeutic potential for better AD treatment in recent years. Qi et al. developed quercetin-loaded exosomes to improve the brain targeting of quercetin and effectively assessed its therapeutic efficacy in a mouse model of AD. Their findings demonstrated that exosomes containing quercetin could alleviate the symptoms of AD by inhibiting cyclin-dependent kinase 5-mediated phosphorylation of Tau and lowering the production of insoluble NFTs (Qi et al., 2020). Wang et al. found that exosomes from curcumin-treated cells can inhibit Tau phosphorylation, reducing AD symptoms *in vitro* and *in vivo* (Wang et al., 2019). Additionally, mesenchymal stem cell-derived exosomes (MSC-Exo) were modified by Cui et al. using a CNS-specific rabies viral glycoprotein (RVG) peptide to improve the potential of exosomes to target the brain when administered intravenously (Cui et al., 2019). RVG-modified MSC-Exo significantly decreases plaque deposition and Aβ levels in the brains of transgenic APP/PS1 mice compared to the group administered unmodified MSC-Exo. Furthermore, the levels of proinflammatory cytokines, such as TNF-α, IL-β, and IL-6, significantly decreased while the levels of anti-inflammatory factors, such as IL-10, IL-4, and IL-13, significantly increased.

### 3.6 Metal NPs

The size of metal NPs is usually between 1 and 100 nm, and they typically have a functional organic molecules shell around a metal core. Presently, pure metals, including gold, silver, copper, zinc, *etc.*, or their oxides make up metal oxide NPs (Jamkhande et al., 2019). For example, cerium and iron oxides make metal oxide NPs, while molybdenum disulfide makes metal sulfoxide NPs. Drug molecules can be coupled to the surface of metal NPs due to their dense spherical carrier structures, large surface area, and potential for drug loading. Additionally, metal NPs are used in targeted drug delivery approaches for CNS diseases because of their excellent biocompatibility, storage stability, ease of synthesis, and few side effects. However, metal NPs are difficult to degrade *in vivo*, and their safety hazards limit their clinical application.

Recently, developed metal-based NPs with higher stability and permeability to tissues like cancer tissues have been used as carriers for photosensitizer drugs. These carriers can be made of gold or magnetically responsive carriers. Nanomaterial formulations have also been used to investigate the targeted delivery of CNS drugs further. Cerium oxide (CeO_2_) NPs exhibit antioxidant, anti-interference, and anti-apoptosis properties. In addition, CeO_2_-NPs existing in the +3 and +4 valence states of cerium have free radical scavenging activity, creating an antioxidant microenvironment and promoting the repair of nerves and other cells (Das et al., 2013). The ability of CeO_2_-NPs with a size <5 nm to mimic catalase and superoxide dismutase through the reversible binding of oxygen atoms and conversion between Ce^3+^ (reducing) and Ce^4+^ (oxidizing) states on their surface has also been demonstrated (Celardo et al., 2011).

The lipophilic cation triphenyl phosphonium can target mitochondria. Studies in the five-familial AD transgenic mouse model have shown that cerium NPs conjugated with TPP can increase mitochondrial concentrations and inhibit neuronal death (Biswas et al., 2012). Another study linked two peptides with the KLVFF sequence (residues 16–20), which corresponds to the hydrophobic core of Aβ and plays a significant role in the formation of the β-sheet structure with ultrasmall gadolinium nanoparticles (AGuIX-NPs). Furthermore, this fragment can also bind to Aβ, affecting the formation of plaques (Lowe et al., 2001).

A magnetic core (such as iron, nickel, cobalt, and their oxides) and a surfactant makeup magnetic NPs combine the benefits of magnetic materials and nanomaterials. Due to their unique superparamagnetic characteristics, magnetic NPs can be guided and deliver drugs to specific areas when exposed to a magnetic field (Materón et al., 2021; Zhang P. et al., 2023). Iron, nickel, and cobalt are frequently used as magnetic materials, iron and iron oxide are the most common materials. Due to its biocompatibility and biodegradability, iron oxide NPs (Fe_3_O_4_ or Fe_2_O_3_) have been used in biomedical research extensively. However, the homogeneity of magnetic NPs is difficult to establish throughout the large-scale synthesis process, and the variation between various production batches is high, which can have major safety problems (Vangijzegem et al., 2019).

### 3.7 Nonmetal inorganic NPs

#### 3.7.1 Carbon nanotubes (CNTs)

CNTs can be divided into single sheets of graphene, also known as single-walled CNTs of 0. 4–2 nm and multi-walled CNTs of 2–100 nm, following the graphene sheet. To achieve drug loading, the wall of nanotubes can not only noncovalently bind to small molecule drugs. Still, it can also physically adsorb small molecule drugs on the surface of carbon nanotubes. Genetic material can be delivered to the nucleus and cells with high efficiency using carbon nanotubes. The few studies on CNTs for AD treatment are still in the early stages and need to be further tested for their ability to cross the BBB (Garalleh, 2018; Mishra et al., 2021). However, CNTs can be used as biosensors with high sensitivity and selectivity to detect Aβ in human serum, particularly Aβ42 and Aβ40 peptides (Chen et al., 2022).

#### 3.7.2 Selenium (Se) NPs

Se is a crucial trace mineral. Se plays structural and enzymatic roles as a component of proteins that contain Se and contributes to various essential physiological functions in the body, including antioxidant defense, immune response, and as a catalyst in thyroid hormone metabolism (Khurana et al., 2019). More importantly, Se can maintain neurotransmitters metabolizing at their normal rate, and as an antioxidant, Se can clear the brain of harmful substances. According to increasing evidence, Se NPs are promising nanoparticle carriers for inhibiting Aβ aggregation and decreasing Aβ neurotoxicity (Zhang Z. H. et al., 2023). Se NPs have a particular affinity for Aβ, which causes Aβ deposition to decrease.

After administering Se to the AD mice model, Song et al. found that the level of β-secretase was downregulated, and the production of Aβ in the brain was significantly decreased (Song et al., 2018). Next, Zhou et al. injected each side of the hippocampus of rats with Aβ40 oligomer to establish AD rat models. Then, they treated AD rats with selenium-doped carbon quantum dot (SeCQD) nanomedicine. They found that the treated rats had shorter escape latencies than rats in the control group during a water maze test, proving that SeCQDs can improve cognitive performance in AD rat models (Zhou et al., 2021).

According to Yin et al., functionalized Se NPs modified with sialic acid on B6 peptides allowed them to cross the BBB, maintain systemic circulation concentration for a long, inhibit Aβ aggregation and reduced cytotoxicity. Furthermore, they constructed a B6-SA-SENP drug delivery system by simulating the structure of selenoprotein using selenium NPs as the core and sialic acid and B6 polypeptide modified on the surface (Yin et al., 2015).

Additionally, selenoproteins, a family of proteins that interact with the C-terminal domain of α-tubulin, are involved in regulating microtubule assembly by including the 21st amino acid, selenocysteine, in their amino acid sequence (Yue et al., 2020). Selenocysteine also interacts with Tau protein, Ca^2+^, and polyamines to reduce the burden of ROS in cells. Thus, the structure and function of microtubules remain protected. Moreover, sodium selenate might improve protein phosphatase 2A activity and dephosphorylate-specific Tau epitopes in SHSY-5Y cells expressing human Tau isoforms to correct neural memory impairment in mice and prevent and reverse memory and motor deficits. Se inhibits the accumulation of aberrant proteins in the brain by decreasing the production of Aβ and Tau phosphorylation. Selenium NPs can therefore be used as carriers to construct functional nano systems, which have potential application value and can overcome the shortcomings of traditional drugs.

## 4 Clinical applications of nanomedicine

The number of patent applications for nanomedicine has significantly increased since the 21st century due to the rapid development of nanotechnology, however, no nanomedicines have yet been approved for treating CNS diseases. Clene Nanomedicine, Inc., a clinical-stage biopharmaceutical company, has developed several nanotechnology-based therapies for CNS diseases and initiated parallel phase II clinical trials (NCT03843710, NCT03536559, NCT03815916, NCT04098406, NCT04626921, and NCT05299658) with the lead asset CNM-Au8^®^. CNM-Au8^®^ is a gold nanocrystal suspension being researched as a disease-modifying treatment for patients with amyotrophic lateral sclerosis, multiple sclerosis, and Parkinson’s disease (Kumar et al., 2022; Vucic et al., 2023). In 2019, Aphios Inc. began a phase II clinical trial (NCT03806478) to evaluate the efficacy and safety of intranasal nanoparticles of APH-1105, in the treatment of AD (Zagórska et al., 2023). However, the outcomes of the current clinical trials of AD nanomedicines have not yielded promising results, and further research is needed to fill the gaps in the industrial field. Although many nanomedicines have been effectively incorporated into clinical practice.

## 5 Challenges and future directions of nanomedicine

Clinical treatments for AD currently focus primarily on symptom management and supplemental brain nutrition. However, these methods offer palliative care without addressing the underlying cause of the disease. The complexity of AD pathology and the challenges associated with drug delivery across the BBB are the primary causes of this limitation. Due to their excellent stability, biocompatibility, degradability, high safety, flexible drug-loading methods, controllable drug release, and surface modifiability, nanomedicine carriers have gained increasing attention. These characteristics make them attractive candidates for targeted therapy in CNS diseases, including AD. Despite the potential advantages, producing nanomedicine commercially is a major challenge. The BBB is the densest barrier in the human body and comprises endothelial cells, pericytes, capillary basement membrane, and astrocytes. It serves as a protective shield, limiting and preventing specific molecules or pathogens from penetrating the brain from the circulatory system. The inability of AD treatment drugs to successfully cross the BBB is a major obstacle (Gao et al., 2017).

**FIGURE 3:**
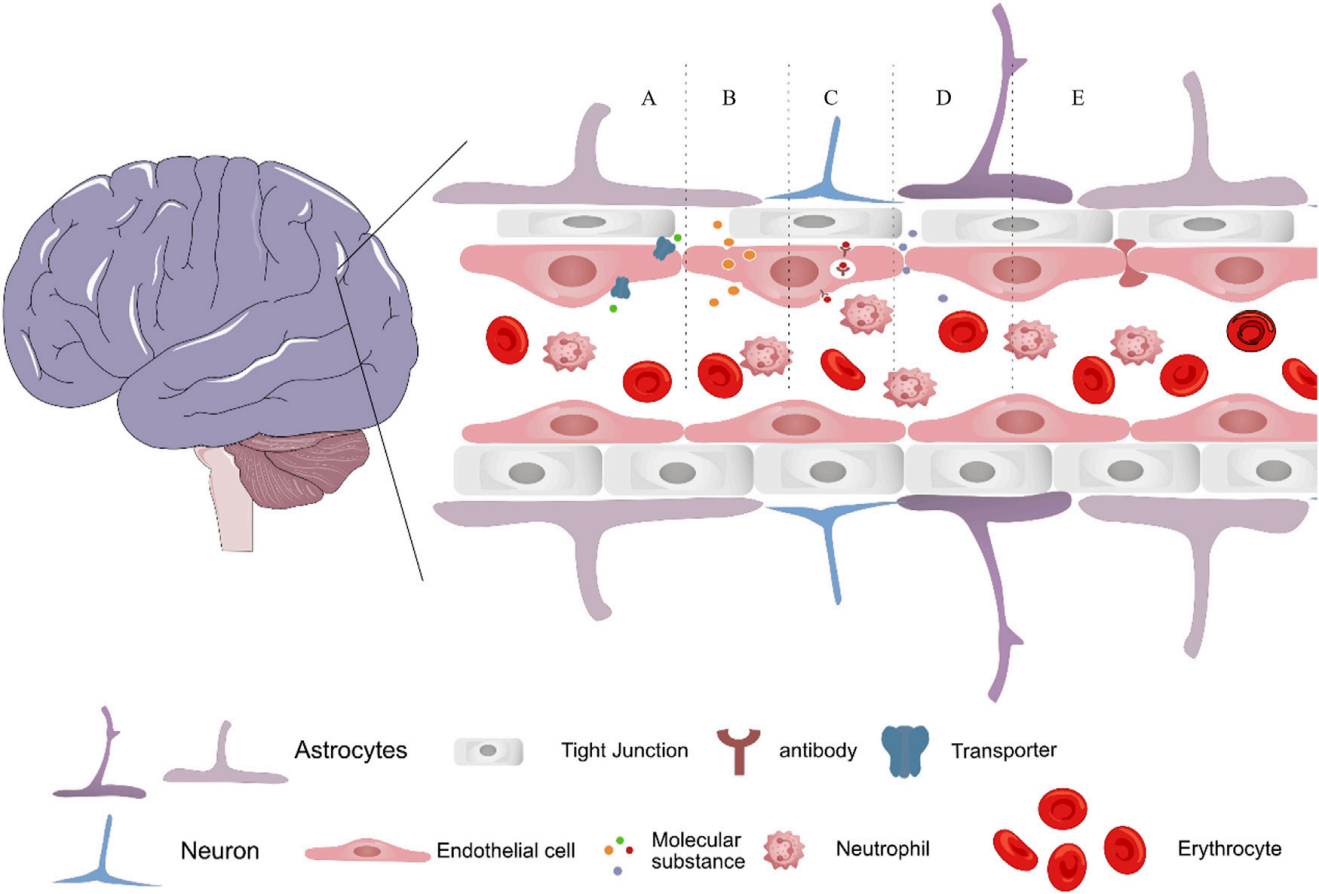
Schematic depicting the transport methods of nanomedicine across the blood–brain barrier, via **(A)** carrier mediated transport, **(B)** adsorptive mediated transport **(C)** receptor mediated transport, **(D)** paracellular transport and **(E)** cell-mediated transport.

Tight junctions between endothelial cells, not large pores, prevent drugs from passing through the BBB. Passively moving across the BBB are small molecules, including water, gases, and other lipophilic compounds. Glucose, amino acids, and most drugs are large, highly charged, highly polar, and highly hydrophilic molecules that specific proteins must transport into cells. Developing nanomedicine that effectively deliver and release therapeutic agents in the brain is challenging but necessary. As shown in Figure 3, the different methods of drug transport across BBB are represented, which include carrier mediated transport, adsorptive mediated transport, receptor mediated transport, paracellular transport and cell-mediated transport. Importantly, transferrin receptor 1 and glucose transporter 1 are expressed at high levels in BBB endothelial cells, and them have been developed as promising targets for CNS diseases (Prakash et al., 2019; Zhou et al., 2020; Li et al., 2023). Future research should focus on eliminating BBB-related obstacles and improving drug delivery systems, and it will progress the study of nanomedicine and pave the way for more effective treatments for AD and other CNS disorders.

The potential for using nanomedicine to treat neurodegenerative diseases is immense. Although few nanomedicines are being studied in clinical trials for AD, there are currently no approved ones. Most nanomedicine research is still preclinical (Kumar et al., 2020). The variety of patients, with each patient displaying a different genetic makeup, neurological function, and characteristics, is one of the major limitations of nanotechnology in treating neurodegenerative diseases. As a result, treating AD cannot be accomplished with a single approach. Furthermore, due to complex manufacturing processes, testing requirements, and regulatory constraints, the development costs of nanomedicine may be higher than those of conventional drugs. Despite these challenges, nanomedicine is still an important topic of study in biomedicine, particularly in cancer treatment, vaccine development, and targeted drug delivery. As a result, several companies and research institutions are investing in developing nanomedicine. The cost-effectiveness of nanomedicine must be thoroughly evaluated during the development and clinical testing process. It is important to strike a balance between these factors and the need to provide more economical treatment options for patients with AD and other neurodegenerative diseases, particularly those in developing countries or with limited financial resources. Moreover, future research should focus on developing customized nanomedicine treatment regimens for each patient’s unique characteristics (Indrasekara et al., 2014). Customized nanomedicine treatments that consider individual differences in physiology, metabolism, and other biological factors are becoming more and more feasible with the growing availability of high-throughput sequencing technology and other types of molecular profiling. Therefore, focusing on customized treatments may significantly impact the development of new and effective nanomedicine therapies. Additionally, customized nanomedicine treatments can reduce healthcare costs by lowering the possibility of unnecessary treatments or adverse effects.

The application of nanomaterials in biomedicine still has many gaps and unresolved issues. Although there have been significant developments in the last decade in the development of nanomaterials for biomedical applications, many challenges still need to be overcome before these materials can be widely applied in clinical settings. Developing novel nanomedicine from the laboratory to the patient’s bedside requires more work. The safety and toxicity of nanomaterials are two major issues. While many NPs demonstrate promising efficacy for drug delivery, imaging, and other biomedical applications, their unique characteristics can also result in unpredictable toxicity and unforeseen effects on biological systems, making it difficult to evaluate their safety using conventional evaluation methods (Wong et al., 2020). Investigating the safety profiles of nanomedicine is challenging for several reasons, including the modifications of nanomedicine, the route of administration, and the complex cellular structure of the brain. The long-term safety of these compounds needs to be carefully evaluated, both *in vitro* and *in vivo,* to minimize the risk of unforeseen outcomes.

The need for standardization in producing and characterizing nanomaterials presents another challenge. For example, it might be challenging to compare the results of various studies or to appropriately evaluate the performance of various types of NPs because there are currently no universally accepted standards for nanomaterials’ characterization and quality control.

Finally, the limited knowledge of the physiological and pathological mechanisms contributing to AD also hinders the development of effective nanomaterial-based therapies. Nanomaterial-based therapies are expected to play a bigger role in treating AD as more research is done to understand the disease’s fundamental biological mechanisms.

## 6 Conclusion

AD is the most prevalent age-related disorder. It is anticipated that as the population ages, the incidence of AD will increase, placing a greater strain on healthcare systems worldwide. Since the underlying causes of the disease are not yet understood, effective therapies are difficult to develop. The main goals of AD treatment are to slow down its progression and improve the patient’s quality of life because there is presently no known cure. Therefore, it is a promising area of research to use nanomedicine to treat AD. The bioavailability of drugs now used to treat AD may be increased by NPs, potentially improving drug delivery. The potential to cross the BBB and target the accumulation of amyloid and Tau proteins, which are main hallmarks of AD pathology, is one advantage of nanomedicine delivery systems. Targeting specific cell types or brain areas using nanomedicine can also increase the specificity of drug delivery and reduce the risk of adverse effects. Controlled drug release can also be attained through nanomedicine, allowing for sustained-release formulations, and optimizing drug dosing. However, there are still risks and unanswered questions associated with using nanomedicine to treat AD, just like with any novel therapy. Since nanomedicine can have unpredictable toxicity profiles, the long-term safety and efficacy of nanomedicine need to be thoroughly evaluated.

In summary, nanomedicine may become a more useful tool for treating and preventing AD with more studies. Using nanomedicine to treat AD has great potential, but more research is required to establish both the safety and effectiveness of these new therapies.
